# Medical News and Miscellany

**Published:** 1886-12

**Authors:** 


### MEDICAL NEWS AND MISCELLANY.

### PERSONAL.

We are much gratified to announce that Dr. T. J. Tyner, the distinguished oculist of San Antonio, has decided to remove to Austin. The position of oculist to the several State institutions here, particularly of the Blind Asylum, made vacant by the death of the lamented Matthis, has been tendered him and been accepted. Dr. Tyner is well known throughout the Southern States as a skillful and successful operator on the eye, and his reputation as an oculist is second to none in the South. He gives up a large and lucrative practice at San Antonio and leaves there under protest of the entire community; but the opportunities presented at the capital—the present and future grandeur of the beautiful city, with its social and other attractions—constitute a temptation too great to be resisted. We congratulate the profession of Austin especially in the acquisition of so acceptable an addition to its ranks. The Doctor will remove on the first of January.

### AN IMPORTANT STEP CONTEMPLATED.

In view of the dangerous proximity of the cholera, and of the urgent necessity for prompt steps to prevent its invasion into the Southern States, Dr. Swearingen, State Health Officer of Texas, has written to Dr. Jos. Holt, President of the Louisiana State Board of Health, at New Orleans, proposing that the health authorities of Mexico be invited to meet with the conference of health officers of the Gulf States and Tennessee, soon to meet in New Orleans, assuming, correctly, no doubt, that should cholera once find entrance into Mexico it would soon spread all over Texas and the South. Dr. Swearingen proposes thus to convert our Inter-State Sanitary Conference into an International organization, thus widening and strengthening its scope and powers. Thus co-operating, it is proposed to make a determined fight, and to bring into requisition all the powers and resources known to modern sanitary science against the dread scourge. Dr. Holt thinks well of the proposition, and having congratulated Dr. Swearingen on the conception of so valuable a suggestion, at once submitted it to the Presidents of the Boards of other States. It is thought the meeting will be called at once, instead of in May, as first contemplated.

### HYPODERMIC MEDICATION.

It will be of interest to the profession to know that hypodermic tablets of all the standard and many of the most recent drugs suitable for hypodermic use, are now prepared without the admixture of any foreign substance whatever, (except when the bulk of a dose is less than 1-12 of a grain,) thus insuring immediate solution and freedom from local irritation. To the list manufactured by John Wyeth & Brother, have been added: Duboisinæ hydrochlor, 1-100 and 1-60 gr.; ditto, with morph sul., gr. 1-8 and 1-4; Hyoscy aminæ sulph. 1-100 and 1-60 gr.; ditto, with morph sul., gr. 1-8 and 1-4. Picro toxini, 1-40 and 1-60. Ditto, 1-80, with strychnia sul. 1-80 gr. Coninæ Hydroborm 1-80 and 1-100 gr.; ditto, with morph sul., gr. 1-6. Curarinæ sulph; Eserinæ sulph., Physostigminæ salicylas, Caffeinæ, Quin, Bi mur et uria, etc., etc. The manufacture of tablets, containing the *exact dose* of these potent drugs, is one of the greatest advances made in pharmacy in years, and one of the greatest services rendered to the medical profession by the manufacturing pharmacist. In this matter John Wyeth & Bro., and Park Davis & Co. have been benefactors. We acknowledge, with thanks, a sample of the tablets of Hyoscyamine from the former house.

---

### DIED.

In Bartlett, Williamson county, Texas, on the 22d ult., Mrs. Mattie Messer Burger, wife of Dr. W. M. Burger, of that place. Deceased was a native of Bell county, Texas.

At Jasper, Texas, September 14, 1886, Mrs. T. M. Stone, wife of Dr. T. M. Stone. The circumstances of Mrs. Stone's death were peculiarly sad. Three weeks after confinement she was attacked with malarial hæmorrhagic fever, and lived only three days. It was the most malignant attack, the Doctor says, that he ever saw. The little infant survived the mother only a few weeks. We tender to our friend and confrere, the bereaved husband and father, our most sincere sympathy.

---

### "PRACTICE"

Is the name of Dr. A. F. Winn's new journalistic venture. It is published at Richmond, Va., the "Sanitary Monitor" being discontinued. We have received the first number, and are pleased with its appearance. Success to the Doctor.

### FOUND AT LAST.

It is claimed (Philadelphia Medical Times) that experiments by Dr. Osler have demonstrated the correctness of the observations of Levaran, Macchiafava, Celli, Colgi, Sternberg and Councilman, and confirm the claim that at last a micro-organism peculiar to malaria has been found. The organism belongs probably to the flaggelate infusoria, and is observed not only in the febrile stage, but also in the apyrexia; and its presence is likely to be of value in diagnosis. The organism, like that of dengue, found by McLaughlin, occupies the blood cell as well as the plasma.

### THE NEW REMEDY FOR TETANUS.

It is claimed by Dr. Carlisle, of Greenville, Ohio (Cleveland Medical Gazette) that urethran, given hypodermically, two-grain doses, increased to four grains in the second hour, partially controlled the convulsive movements in a case of traumatic tetanus to which he was called five days after the appearance of the malady. The Doctor thinks if it had been given earlier it might have been more successful. He recommends physicians to be prepared with a supply of the alkaloid (which is soluble and non-irritating) to meet cases of strychnine poisoning, urethran being said to be the physiological antidote to that drug. Dr. C. thinks urethran is the coming remedy for tetanus, and that it is certainly worthy of a more extended trial in that condition, especially as all known remedies have failed to control it.

### QUARANTINE.

The Governor of Texas has very promptly quarantined against South America, and our health authorities will take every precaution to prevent the cholera from getting a foot-hold in this State, as it did seventeen years ago.

### MARRIED.

ROBERTSON–LEPPOLD—At Lockhart, Texas, November 10, 1886, at the home of the bride's parents, Dr. H. S. Robertson of Cedar Creek, Texas, to Miss Lizzie Leppold.

LAW–BARTON—At the residence of the bride's mother, in Salado, Sunday, November 21, Dr. Jarrett D. Law and Miss Milda Barton. The groom is associated with Dr. Bob Barton in the practice of medicine, and is universally liked. The bride is a woman of culture and quite popular. THE JOURNAL extends its heartiest congratulations.

